# nocoRNAc: Characterization of non-coding RNAs in prokaryotes

**DOI:** 10.1186/1471-2105-12-40

**Published:** 2011-01-31

**Authors:** Alexander Herbig, Kay Nieselt

**Affiliations:** 1 Center for Bioinformatics Tübingen, University of Tübingen, Sand 14, 72076 Tübingen, Germany

## Abstract

**Background:**

The interest in non-coding RNAs (ncRNAs) constantly rose during the past few years because of the wide spectrum of biological processes in which they are involved. This led to the discovery of numerous ncRNA genes across many species. However, for most organisms the non-coding transcriptome still remains unexplored to a great extent. Various experimental techniques for the identification of ncRNA transcripts are available, but as these methods are costly and time-consuming, there is a need for computational methods that allow the detection of functional RNAs in complete genomes in order to suggest elements for further experiments. Several programs for the genome-wide prediction of functional RNAs have been developed but most of them predict a genomic locus with no indication whether the element is transcribed or not.

**Results:**

We present NOCORNAc, a program for the genome-wide prediction of ncRNA transcripts in bacteria. NOCORNAc incorporates various procedures for the detection of transcriptional features which are then integrated with functional ncRNA loci to determine the transcript coordinates. We applied RNAz and NOCORNAc to the genome of *Streptomyces coelicolor *and detected more than 800 putative ncRNA transcripts most of them located antisense to protein-coding regions. Using a custom design microarray we profiled the expression of about 400 of these elements and found more than 300 to be transcribed, 38 of them are predicted novel ncRNA genes in intergenic regions. The expression patterns of many ncRNAs are similarly complex as those of the protein-coding genes, in particular many antisense ncRNAs show a high expression correlation with their protein-coding partner.

**Conclusions:**

We have developed NOCORNAc, a framework that facilitates the automated characterization of functional ncRNAs. NOCORNAc increases the confidence of predicted ncRNA loci, especially if they contain transcribed ncRNAs. NOCORNAc is not restricted to intergenic regions, but it is applicable to the prediction of ncRNA transcripts in whole microbial genomes. The software as well as a user guide and example data is available at http://www.zbit.uni-tuebingen.de/pas/nocornac.htm.

## Background

In the past few years non-coding RNAs (ncRNAs) have been increasingly recognized to be involved in a variety of biological functions, especially gene regulation [1-4]. Several classes of regulatory or catalytic ncRNAs have been discovered. Some of them such as miRNAs or snoRNAs only occur in eukaryotes [5]. In prokaryotes ncRNAs are of interest, for example because of their potential role in pathogenicity [6-9], their specialized housekeeping functions, or their involvement in various stress situations [10-12]. A special class of ncRNAs are antisense RNAs (asRNAs), which are located antisense to protein-coding genes, and which act as putative regulators via base pairing interaction with their antisense gene [13].

Several experimental techniques are used to identify bacterial ncRNAs [14-16]. However, these methods are laborious and expensive, especially if a large number of elements is analysed. Next-generation sequencing techniques have been applied to analyse complete transcriptomes of bacteria under various conditions, which also led to the discovery of numerous novel ncRNA transcripts [17-21]. However, ncRNAs that are not expressed under the specific conditions of the experiment will not be detected.

Therefore, computational predictions of genomic loci which contain a functional ncRNA are usually conducted to either complement the analyses of experimental data or to suggest candidates for further experiments [22]. A plethora of computational methods for the prediction of functional ncRNAs have been developed (see [23] for a review). Most of them exploit the structural conservation and the higher structural stability of ncRNAs [24-29]. Other methods are based on sequence clustering [30], graph processing [31] or various machine learning approaches [32-35]. The aim of most of these methods is to identify regions that contain functional ncRNAs. However, most of the programs do not directly assess the question if the predicted ncRNA is transcribed or if it contains an untranscribed RNA motif. Furthermore, when applied to large genome alignments, programs such as RNAz use a window-based approach, so that the boundaries in particular of ncRNA transcripts are often imprecise. Another problem is the correct determination on which strand the ncRNA resides.

To address these problems some approaches, e.g. SIPHT, sRNAFinder, sRNAPredict, or sRNAscanner integrate heterogeneous data such as transcription start sites (TSS) and transcription termination signals [36-39]. In principle, known transcription factor binding sites (TFBS) could be used to predict the 5' start of ncRNA transcripts. However, the number of different transcription factors varies between species. In *Streptomyces coelicolor*, for example, there are 65 sigma factors [40] and for most of them a sequence pattern of their specific binding site is not known. Furthermore, since genome-wide TFBS annotations are often not available, a more general model is needed.

Here, we introduce NOCORNAc (**no**n-**co**ding **RNA c**haracterization), a Java program for the prediction and characterization of ncRNA transcripts in bacteria. NOCORNAc takes the coordinates of putative ncRNA loci as input and annotates them with transcriptional features to conduct strand-specific transcript predictions. While previous computational approaches to identify non-coding RNAs in bacterial genomes have restricted the analysis to intergenic regions [41-43], our approach is not limited to intergenic regions but also applied to predict cis-encoded asRNA transcripts. For the detection of the transcript's 3' end NOCORNAc integrates the program TransTermHP [44] to predict Rho-independent terminator signals. The 5' start is predicted by the detection of destabilized regions in the genomic DNA. For this purpose we implemented the so-called SIDD model [45], which has been shown to be applicable to the detection of promoter regions in microbial genomes [46,47]. Therefore, NOCORNAc does not have to rely on information about known TFBS. The putative transcriptional features are then combined to classify ncRNA loci into either being an ncRNA transcript or not. For ncRNAs that are classified as transcripts the strand is automatically specified, and its boundaries are derived from the SIDD sites and the Rho-independent transcription termination signal. Those loci that are classified not to be a transcript might be false positive predictions or they contain cis-regulatory motifs. For the latter, NOCORNAc incorporates other functionalities for the further analysis of the ncRNA loci such as the search for known RNA motifs from the Rfam database. Furthermore, NOCORNAc provides methods for the prediction of RNA-RNA interactions between ncRNAs and mRNAs. All results can be studied in detail in NOCORNAc's integrated interactive R environment.

We used RNAz [24,25] and NOCORNAc to perform a genome-wide computational screen for ncRNAs in *Streptomyces coelicolor*. Predictions and experimental validations of ncRNAs of *S. coelicolor *have been previously reported [48,49]. All of the studies have restricted their search to intergenic regions. We used RNAz for the detection of ncRNA loci in *S. coelicolor *including the prediction of cis-encoded asRNA loci [50]. These results were used to design a custom expression microarray targeting asRNA regions in the genome of *S. coelicolor *in addition to protein-coding genes and intergenic regions [51]. In the trans-national Systems Biology consortium SysMO/STREAM we used this array to generate high resolution time-series gene expression data for *S. coelicolor *grown in fermenters [52]. In the current study we use these data to validate predicted ncRNA transcripts as well as to compare expression profiles of asRNA transcripts predicted by NOCORNAc with their sense partner gene.

## Methods

### Identification of transcription termination signals

To predict Rho-independent termination signals we integrated the program TransTermHP [44]. This tool detects stem-loop motifs in whole genomes and scores them with respect to their potential ability to act as transcription terminators. The scoring of each motif is done for three parts, the stem, the loop and the tail, which is the single-stranded region following the 3' end of the stem-loop. The stem is scored with respect to its size and GC-richness. The loop is scored by its size and the tail is also scored with respect to nucleotide composition as, for example, a large number of AU-base pairs in this region promotes the dissociation of the transcript due to the lower stability of such base pairs. The three scores are then combined to a single confidence value for each predicted terminator.

### Identification of promoter regions

For the identification of promoter regions we implemented the so-called SIDD model (Stress Induced Duplex Destabilization) [45]. The approach not only considers the thermodynamic stability of the base pairs on a dinucleotide level, but it also takes into account the torsional energy that is needed to unwind the helix as well as the influence of superhelical stress.

Using this model, a SIDD profile is calculated for a stretch of genomic DNA. For each position it denotes the expected additional free energy needed to separate the base pair at that position. To calculate this profile for a region of length *n *the model has in theory to consider all 2*^n ^*possibilities to separate the helix in that region. As this would be too time-consuming, only biologically plausible separation patterns are taken into account, which results in a worst case runtime complexity of *O*(*n*^3^). Partition functions are used to calculate the SIDD value for each position. For further details we refer to the original publication [45].

We implemented the model as described in [45]. To maximize memory and runtime efficiency only native Java arrays (int, double) were used. The calculation of the SIDD profile for a complete prokaryotic genome is accomplished by a sliding window. The SIDD calculation for the genome of *S. coelicolor *was conducted using a window size of 10,000 nt and a step size of 1,000 nt. Therefore, each position is contained in 10 windows and thus 10 values are calculated. We summarize them using a weighted average, where windows in which the position is near the center of the window get a higher weight than windows in which the position is near the border. This approach has been suggested in [45]. The calculation of the SIDD profile for the genome of *S. coelicolor *takes about 48 h on a single core CPU and needs less than 512 MB memory. If more than one core/processor is available NOCORNAc calculates the window profiles during the sliding window approach in parallel. Therefore, the procedure takes only some hours on a modern multicore system.

### Prediction of ncRNA transcripts

All ncRNA loci are annotated with the transcriptional features that have been predicted at their locus. This annotation is used to decide if a locus potentially contains a transcript, or if it might be an untranscribed RNA motif. For the transcript prediction step, terminator signals and SIDD sites are combined. This not only allows the specification of the strand of the potential ncRNA transcript, but also the more exact delineation of the specific element. First, SIDD sites associated with predicted ncRNA regions are considered. The prediction process is applied to each SIDD site of the predicted ncRNA region, and for each site it is applied to both strands as SIDD sites are not strand-specific. Taking a SIDD site as a start point, the predicted transcript is extended in the direction of the currently processed strand. The end point is either the first *high confidence *terminator, which is a terminator signal with a confidence value of at least 76 [44] or, if all signals have a lower value, the terminator with the highest confidence value which is found downstream of the SIDD site. If no terminator signals are found at all, the transcript is extended until the end of the predicted ncRNA region is reached, but only if the SIDD site, which has been taken as the start point, cannot belong to a protein-coding gene. Overlapping transcripts, which are located on the same strand, are joined after the prediction procedure. Furthermore, in the case that transcripts are predicted on both strands and the two predictions overlap, only the transcript with the better terminator confidence value is kept. The other prediction is trimmed by assigning an alternative terminator signal that is closer to the SIDD site, so that the two transcript do not overlap any more. If this is not possible, the transcript with the weaker terminator signal is discarded.

### Searching the predicted elements for motifs from the Rfam database

We integrated a functionality to automatically search ncRNA loci for ncRNA motifs that are stored in the Rfam database [53]. For this task we incorporated the programs cmsearch[54] and Erpin[55]. Using a set of Rfam seeds, that can be retrieved from the database, motif descriptors are generated for both programs. By default motifs are searched with Erpin. However, for certain motifs it is not possible to setup an Erpin search automatically. In these cases cmsearch is used instead. If a multicore system is used, the procedure is parallelized.

### Interactive R environment

Parts of the data structure are provided within an interactive R[56,57] environment, allowing the user to perform a variety of statistical analyses to the results as well as to visualize them. This also includes some basic sequence operations by which the user can, for example, extract genomic sequences of previously selected features like predicted ncRNA-regions. Furthermore, each predicted ncRNA transcript can be visualized in the context of all detected transcriptional features by the use of a predefined plotting function. It is also possible to perform individual RNA-RNA interaction predictions between any elements that are contained in the environment.

### nocoRNAc

**Figure 1:**
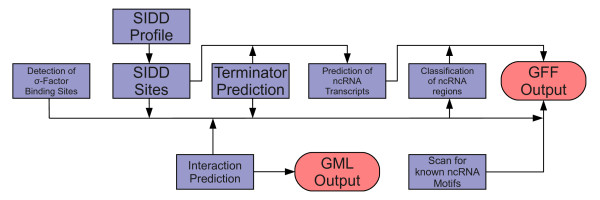
**Schematic representation of nocoRNAc's workflow**. Schematic representation of NOCORNAc's workflow. The sequence of the target genome, locus information on coding genes and predicted ncRNA regions are taken as input. The promoter region prediction includes the detection of TFBS (regular expression) and of destabilizable regions (SIDD sites). The latter together with predicted transcription terminators are used to predict ncRNA transcripts. All results are used to classify and annotate the ncRNA regions. The combined output is a GFF file.

The described methods are combined in the Java program NOCORNAc. NOCORNAc reads coordinates of predicted ncRNA loci in GFF or simple tabular format. Coordinates of protein-coding genes have to be provided in PTT format. In addition, the genomic sequence is read from a FASTA file. The program is started with a single command and all integrated procedures are applied automatically in the form of a pipeline. A schematic overview of NOCORNAc's workflow is shown in Figure 1. After the SIDD profile has been calculated it is used to predict SIDD sites, which are - together with the results of the terminator prediction - assigned to the ncRNA regions. These features are then used to predict ncRNA transcripts. If the user provides sequence patterns for transcription factor binding sites in the form of regular expressions, they are used to scan the genome for the respective binding sites and to annotate the ncRNA loci with the resulting hits. Optionally, the loci can also be scanned for RNA motifs contained in the Rfam database.

All intermediate data is stored in the project folder. Therefore, it is possible to access specific feature information manually (e.g. predicted terminators or the SIDD profile). In addition, time-consuming procedures, like the SIDD calculation, only have to be performed once, as NOCORNAc reads already produced results, if available. NOCORNAc can also perform RNA-RNA interaction predictions utilizing IntaRNA [58]. The user can specify the elements that will be included in the analysis. The interaction prediction can also be started in NOCORNAc's R environment.

There are different ways to access the generated results. On the one hand all results are condensed in a single GFF file, which can be viewed by standard genome browsers. In addition, some general statistics are written to standard out, e.g. the number of ncRNA loci provided as input or the number of predicted ncRNA transcripts. On the other hand the user can access the data quite specifically by using NOCORNAc's R environment. This is especially useful for the detailed investigation of subsets of the data or certain predicted elements that are of particular interest.

### Genome-wide functional ncRNA prediction in S. coelicolor

For the genome-wide prediction of ncRNA loci we used the program RNAz[25], which takes a sequence alignment as input and classifies it as 'RNA' or 'OTHER'. The prediction approach of RNAz is mainly based on two principles: The first principle exploits the fact that functional ncRNAs usually exhibit a significantly more stable structure than non-functional ncRNA sequences. This is at least true if the function is based on a certain structure, which is, for example, not the case when dealing with protein-coding RNAs. The second principle is based on the so-called structure conservation index (SCI), which measures the structure conservation between the aligned sequences. It is assumed that the structure of functional RNAs is usually more conserved between related species than the structure of other sequences. The final classification is accomplished by an SVM that has been trained on the RNA families contained in the Rfam database.

As RNAz needs a multiple sequence alignment as input, we aligned the genomic sequences of *S. coelicolor *[RefSeq:NC_003888.3], *S. avermitilis *[RefSeq:NC_003155.4] and *S. griseus *[RefSeq:NC_010572.1] using the genome alignment software mauve (version 2.3.1) [59,60]. The resulting alignment was converted to maf format. To be able to detect ncRNAs of different size we performed several runs of RNAz with different settings for the window size, i.e. 60, 80, 100, 120 and 160 nt. The step size was set to 20 nt. All windows that did not contain sequence information for all three species (e.g. if there is a large deletion in one of the genomes) were not considered in further analyses. After the application of RNAz, overlapping windows that had been classified as 'RNA' were joined to predicted ncRNA loci. As a threshold an SVM P-value of 0:5 was used. The predicted ncRNA loci were then used as input for NOCORNAc.

### Microarray analyses

For expression studies we used a custom-designed microarray, which contains 226,576 perfect match oligonucleotide probes interrogating 8,205 protein-coding regions, 10,834 intergenic regions with a tiling approach, and 3,672 regions antisense of protein-coding genes in the genome of *Streptomyces coelicolor *[51]. In a previous study this array has been used to produce high resolution time-series expression data for the model organism *Streptomyces coelicolor *grown during submerged batch fermentations [52]. *S. coelicolor *M145 *wt *was cultivated under phosphate limited conditions to monitor the effect of this limitation on the expression of protein-coding genes. Phosphate was depleted at 35 h after inoculation. Samples were taken at 32 time points, covering the interval from 20 h to 60 h after inoculation.

In order to profile the expression of the predicted ncRNA transcripts we aligned all probes of the chip to the predicted ncRNAs. All predicted transcripts that have at least 4 probes completely overlapping their genomic locus were added as a new probeset to the Affymetrix CDF descriptor of the chip. Normalized expression values were generated using RMA as described for the protein-coding genes [51,52]. Expression profile analysis and visualization was done using Mayday [61].

## Results

### Genome-wide detection and classification of ncRNAs

The alignment of the genomes of *S. coelicolor*, *S. avermitilis *and *S. griseus *produced by Mauve after pre-processing by rnazWindow covered 34.6% of *S. coelicolor*'s genomic sequence. Starting from the genome alignment, using a desktop PC with 4 GB RAM the prediction of ncRNA loci with RNAz needed 24 hours, the computation of the SIDD profile took 48 hours, and the prediction of terminators using TransTermHP was finished after 30 s. Finally, NOCORNAc used another 3 s for the transcript models and generation of the results.

RNAz predicted 4,707 ncRNA loci (P-value ≥ 0.5) for the reference organism *S. coelicolor*. Of these loci NOCORNAc annotated 2,358 with a Rho-independent terminator signal and 2,237 with a SIDD site. Combining these annotations NOCORNAc predicted 843 ncRNA transcripts of which 653 are located anti-sense to a protein-coding region. 10 predicted transcripts are partially overlapping a coding region in sense direction. 180 predicted transcripts are located in an intergenic region. The comparison of those elements to annotated ncRNAs revealed that 96 map to known ncRNA genes like rRNAs or tRNAs. Thus 84 putative novel intergenic ncRNA transcripts were predicted by NOCORNAc.

A GFF file containing all predicted elements is provided as additional file 1. In addition, a table listing all predicted ncRNA transcripts together with supplementary information is provided as additional file 2.

**Figure 2:**
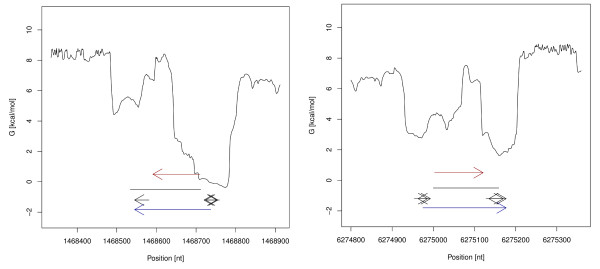
**Transcription feature plots of ncRNA transcripts**. Transcription feature plots of ncRNA transcripts predicted by NOCORNAc (blue arrows) covering annotated ribosomal RNAs (red arrows). The SIDD profile of the genomic region is drawn as a black graph (related scale on the y-axis). The coordinates of the genomic region are denoted on the x-axis. The ncRNA locus predicted by RNAz is shown as a black line. Predicted Rho-independent terminator signals are depicted as short black arrows. NOCORNAc considers the properties of the predicted transcription features (free energy value of SIDD sites; confidence value for terminators) and not only their position to predict the strand.

After a run of NOCORNAc the results can be accessed in the integrated R environment. One feature is the generation of plots for a given genomic region, in which the transcriptional features together with the predicted ncRNA and other annotations are visualized (see Figure 2). We will demonstrate NOCORNAc's procedure using examples of known 5 S ribosomal RNAs. In Figure 2 this is visualized in the context of the transcriptional features used for the prediction. The predicted transcripts start at a significant drop in the SIDD profile (SIDD site) and extend to the best detected terminator signal downstream. Note that there are additional SIDD sites at the other end of the predicted ncRNA loci, and there are also terminator signals that could be used to predict transcripts on the other strand in combination with these SIDD sites. However, NOCORNAc discards the transcript with the weaker signals in such a case and in the depicted situations the strand of the ncRNA transcripts was correctly predicted. It can be seen that the predicted transcripts are longer than the actual annotated ribosomal RNAs. For the transcript prediction we include the complete SIDD site for the 5' start of the transcript, since a precise transcription start site cannot be deduced from the SIDD site. For the 3' end we consider a Rho-independent terminator signal to be part of an RNA transcript, though the conserved structure of the functional RNA that is transcribed might end further upstream.

**Figure 3:**
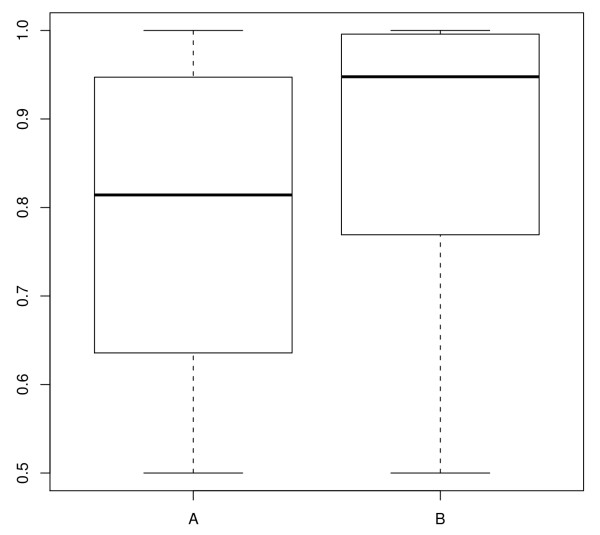
**Boxplots of RNAz P-value distributions**. Boxplots of RNAz P-value distributions of predicted ncRNA loci without transcript prediction (A) and regions for which NOCORNAc predicted an ncRNA transcript (B).

To investigate if there is a relation between the P-value of an ncRNA region predicted by RNAz and the probability that NOCORNAc predicts a transcript in this region we created two sets of ncRNA regions. One set (A) contained only regions without a predicted transcript and one set (B) contained only regions for which NOCORNAc predicted an ncRNA transcript. The comparison of the two P-value distributions revealed that regions containing an ncRNA transcript predicted by NOCORNAc tend to have a better P-value than other regions (Figure 3). More than 60% of all predicted ncRNA transcripts belong to a region whose P-value exceeds 0.9. To verify that the two distributions differ significantly a one-sided two-sample T-test has been conducted, which resulted in a p-value of 6.66*e *- 49. If stricter thresholds are used for the transcript prediction (SIDD site's free energy value ≤4 kcal/mol; terminator confidence ≥76) even 90% of the transcripts were predicted for loci with P-values > 0.9.

To assess the overall sensitivity and specificity of our genomic RNAz screen and of NOCORNAc we compared the predicted ncRNA regions and the predicted ncRNA transcripts to all annotations of ncRNAs in *S. coelicolor *that can be found in the NCBI database (see a summary of these results in table 1).

**Table 1:** Comparison of predicted ncRNA loci and transcripts to annotation from NCBI and Rfam for S. coelicolor

annotated ncRNAs	RNAz locus	predicted transcript nocoRNAc [correctness %]	predicted transcript SIPHT [correctness %]
21 ncRNA genes	21 (100%)	16 (76%)	13 62%)
65 tRNAs	57 (88%)	30 (53%)	1 (2%)
28 cis-regulatory motifs	17 (61%)	1 (94%)	2 (93%)

The first column contains the numbers of annotated elements for 3 types of ncRNAs in *S. coelicolor*: ncRNA genes (without tRNAs) and tRNAs from NCBI as well as cis-regulatory motifs from Rfam. The second column indicates the number of elements for which RNAz predicted an ncRNA locus (strand-unspecific). Columns 3 and 4 indicate the number of annotated elements predicted to be an ncRNA transcript (strand-specific) by NOCORNAc and SIPHT, respectively.

**Figure 4:**
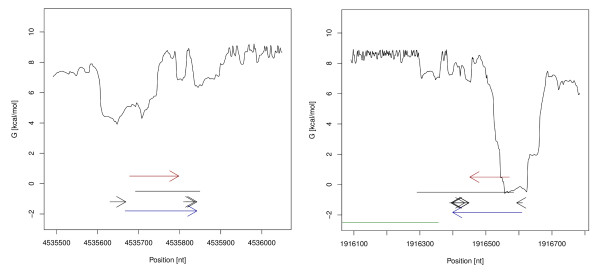
**Transcription feature plots of predicted ncRNA transcripts**. Transcription feature plots of predicted ncRNA transcripts (blue arrows) covering annotated ribosomal RNAs (red arrows). For a detailed legend see figure 2. In the first example the RNAz prediction is shorter than the annotated ncRNA (left), while it is much longer in the second example (right). In both cases the prediction of the transcript boundaries were improved by NOCORNAc.

For all 21 annotated ncRNA genes excluding tRNAs an ncRNA region was predicted by RNAz. 76% of these were correctly classified as an ncRNA transcript by NOCORNAc. When comparing annotations to ncRNA transcripts predicted by NOCORNAc the strand information is taken into account as NOCORNAc also predicts the strand of the transcript. Using standard parameters NOCORNAc does not predict transcripts on different strands that overlap each other. If this was allowed, at 19 of the 21 annotated ncRNA gene loci an ncRNA transcript was predicted. Thus, for 3 annotated ncRNAs the strand-specification was done incorrectly by NOCORNAc. Of the 16 detected ncRNA genes 7 have very strong transcriptional signals, i.e. a SIDD site with a free energy value <4.0 kcal/mol and a predicted terminator with a confidence value exceeding 75, which is regarded as the threshold for high confidence terminators by the authors of TransTermHP [44]. In 3 of these cases the RNAz prediction was shorter than the annotated ncRNA, which could be improved by NOCORNAc. In 2 other cases the predicted ncRNA locus was much longer, while the transcript prediction of NOCORNAc was able to delineate the actual coordinates of the ncRNA gene more precisely (see Figure 4 for examples). Of the 65 annotated tRNA loci 57 were predicted by RNAz, of which 30 were correctly predicted as ncRNA transcripts, including strand-specification, by NOCORNAc. An additional 4 tRNAs were also predicted as ncRNA transcripts, but located on the wrong strand. We also compared our predictions to annotated cis-regulatory elements that can be found in the Rfam database (10.0) and which are not transcribed independently from an mRNA. Here we expect NOCORNAc to classify those loci not to be transcripts. For 17 of 28 cis-regulatory elements an ncRNA locus was predicted. Only one element was predicted as a transcript by NOCORNAc, thus a correctness of over 90% was achieved here.

For a further assessment of NOCORNAc's performance we also applied SIPHT to the genome of *S. coelicolor*. SIPHT is a computational pipeline for the prediction and annotation of bacterial non-coding RNAs [36]. This program predicts ncRNAs restricted to intergenic regions. However, it also as NOCORNAc uses sequence and structure conservation, Rho-independent transcription terminators and, if available, transcription factor binding sites. Therefore, we deemed it to be most comparable with NOCORNAc. We used the SIPHT web interface with standard parameters. Altogether SIPHT reported 391 intergenic ncRNA transcripts. We then also compared these results to the annotated elements. As for nocoRNAc the strand information of the predictions is taken into account. A summary of both comparisons is given in table 1. SIPHT only predicts two cis-regulatory elements incorrectly to be ncRNA transcripts, while NOCORNAc only predicts one such element falsely. SIPHT finds 14 out of 86 known ncRNAs, while NOCORNAc predicts 46 of these 86 correctly. In particular, SIPHT has only predicted one tRNA of the 65 annotated tRNAs, while NOCORNAc's sensitivity for this class of ncRNAs is over 50%.

### Time-series expression analysis of predicted ncRNA transcripts

For 403 of the 843 predicted ncRNA transcripts we measured the expression profile at 32 time points along the growth curve of *S. coelicolor *under phosphate limited conditions [52] using a custom design Affymetrix microarray [51]. 92 elements are located in an intergenic region, of which 47 are putative novel ncRNA transcripts. First, we wanted to assess for how many predicted ncRNA transcripts expression was detected. As a threshold for minimal expression we choose the first quartile of the expression value distribution of the protein-coding genes. Using this threshold we found 317 of the 403 measured ncRNA transcripts to show expression in one time point at least. After variance filtering (regularized variance ≥0.025) we considered 71 of these predicted transcripts to be differentially expressed across the time-series.

**Figure 5:**
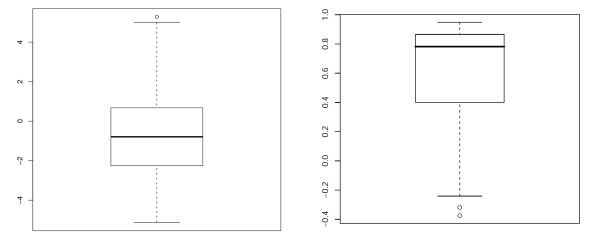
**Boxplots of expression differences and correlations**. Left: Boxplot of average expression profile differences of predicted asRNAs and their respective protein-coding genes. A negative value indicates a higher expression level of the coding gene. (*x *= asRNA; *y = *protein $d(x,y)=(\sum_{i=1}^{n}x_{i}-y_{i})/n).$ Right: Boxplot of expression profile correlations of predicted asRNAs with a variant expression profile and their respective protein-coding gene.

We then compared the absolute expression levels of protein-coding genes and their predicted antisense RNAs. For this we calculated the average per time point expression value difference of the expression profiles. All 235 predicted asRNAs for which expression was detected and the respective coding genes were included in this calculation. The resulting distribution is visualized as a boxplot in Figure 5 (left). In about 35% of the cases the predicted antisense RNA has a higher expression level than the respective coding gene.

For all 47 asRNAs with a variant expression profile we computed the expression profile correlation with their respective antisense genes. A boxplot of the distribution is shown in Figure 5 (right). The median pairwise correlation is 0.78 and about 75% of the pairs show an expression profile correlation above 0.4. The remaining 25% tend to have a low correlation or even a slight anticorrelation.

**Figure 6:**
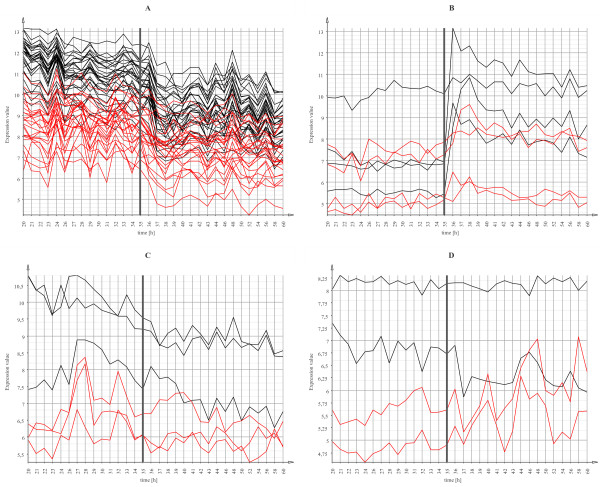
**Expression profile plots of 4 clusters of asRNAs**. Expression profile plots of 4 clusters of asRNAs (red) and their protein-coding genes (black), which resulted from an unsupervised expression profile clustering. The time point of phosphate depletion is indicated by a grey vertical line.

In the next step we conducted an unsupervised expression profile clustering of the 47 variant asRNAs (Figure 6). Most of them show an expression profile that reacts to the depletion of phosphate in the medium at 35 h after inoculation. 24 predicted ncRNA transcripts are downregulated after that time point (Figure 6A). The antisense genes also show a clear downregulation after 35 h together with their predicted antisense RNAs. Most of them encode ribosomal proteins. 4 predicted asRNAs show an upregulation at the time of phosphate depletion together with their antisense genes (Figure 6B). Again the expression of genes and their predicted antisense RNAs is highly correlated. Among the genes in this group are the polyphosphate kinase Ppk (SCO4145) and the phosphate binding protein PstS (SCO4142), for which it has been shown that they are regulated by PhoP, a regulator responding to phosphate limitation [62].

The genes in clusters C and D of Figure 6 encode developmental proteins involved in chromosome replication or RNA synthesis, for example. They also show a downregulation that is probably triggered by the depletion of phosphate.

**Figure 7:**
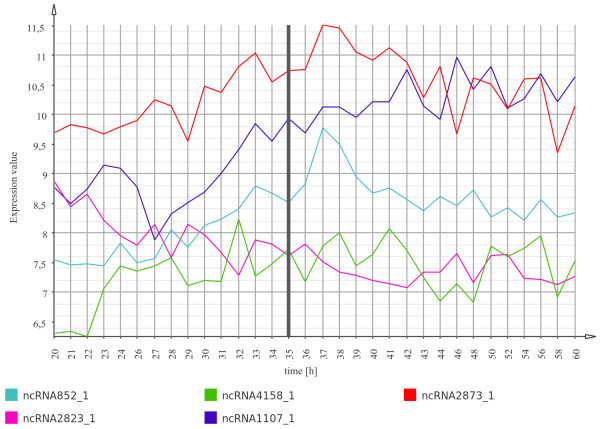
**Expression profile plot of predicted intergenic ncRNA transcripts**. Expression profile plot of predicted ncRNA transcripts that are located in intergenic regions and that show a variant expression profile. The time point of phosphate depletion is indicated by a grey vertical line.

In addition to the asRNAs we were able to profile the expression of 92 predicted intergenic ncRNA transcripts. Using the same expression threshold as for the asRNAs, 82 of them are considered transcribed, of which 38 are putative novel transcripts. Expression profiles of some predicted ncRNAs showing a variant expression pattern are depicted in Figure 7. Interestingly, ncRNA852_1 and ncRNA2873_1 show a quite similar expression pattern, which appear to be up-regulated after phosphate depletion.

A table containing expression data for all predicted ncRNA transcripts that have been measured is provided as additional file 3.

## Discussion

We presented NOCORNAc, a program for the genome-wide prediction and characterization of ncRNA transcripts. As input NOCORNAc uses predicted loci containing functional ncRNAs. In our study we used RNAz to predict the coordinates of ncRNA loci. However, NOCORNAc is not limited to data generated by RNAz. Loci can also be predicted using other programs like QRNA [26] or EvoFold [27], for example. In addition, also loci from an RNA-seq experiment or that resulted from manual annotation can be taken as input. As NOCORNAc itself runs on a single genome, the loci also do not have to be generated by a comparative approach. Nevertheless, we plan to integrate comparative methods in order to assess the confidence of the predicted transcriptional features that are used for transcript prediction in more detail.

For the classification which of the loci contain transcribed ncRNAs and to further characterize the loci, NOCORNAc combines different methods for the prediction of transcriptional features. We demonstrated that NOCORNAc is applicable to predict ncRNA transcripts in the context of previously detected ncRNA loci including strand-specification.

Most bacterial ncRNAs are transcribed from their own promoters, and transcription most often terminates at a strong Rho-independent terminator. For the detection of the latter we integrated TransTermHP. One of the main advantages of this approach is that it is very fast, and the method can define the 3' end of a transcript quite precisely. However, the model fails for transcripts whose transcription is terminated Rho-dependently. Therefore, NOCORNAc can only be applied to those bacteria where Rho-independent termination is the major mechanism of transcription termination. One of the problems is the choice of a threshold value for a terminator signal. The authors of TransTermHP recommend to use 50 [44], which is implemented as default in NOCORNAc. During transcript prediction all terminator signals detected in the genomic context of an ncRNA locus are considered and our model chooses the best one with regards to the local context and the confidence value. This, however, does not rule out that false positive predictions still remain.

For the prediction of transcription start sites NOCORNAc integrates the SIDD model. Although SIDD sites do not specifically occur at transcription start sites [63,64] and their association with promoter regions has mainly been shown for protein-coding genes [46,47], we were able to show that this approach is also applicable to ncRNA genes. When comparing to the 21 known ncRNAs in *S. coelicolor*, we found 15 with a clear SIDD site. Though the energy value for SIDD sites of predicted ncRNAs were generally weaker than for protein-coding genes, the signal is still specific enough to detect their promoter region.

Furthermore, we also showed that there is a clear correlation of the presence of transcriptional features for an ncRNA locus and its RNAz P-value. This indicates that the transcriptional features that are used for the transcript predictions can be used to further increase the confidence of predicted ncRNAs.

NOCORNAc does not predict long ncRNAs such as 23S ribosomal RNA. For such ncRNAs the transcript prediction is more difficult because RNAz is not able to detect a single contiguous locus for such long transcripts. Several loci scattered over the respective regions are predicted instead. This makes it very difficult to predict transcripts correctly as NOCORNAc performs transcript prediction in the context of these ncRNA loci. Thus the quality of NOCORNAc's transcript predictions significantly depends on the quality of the loci provided as input. Nevertheless, we have shown that NOCORNAc can to some extent compensate inaccurate locus predictions.

**Figure 8:**
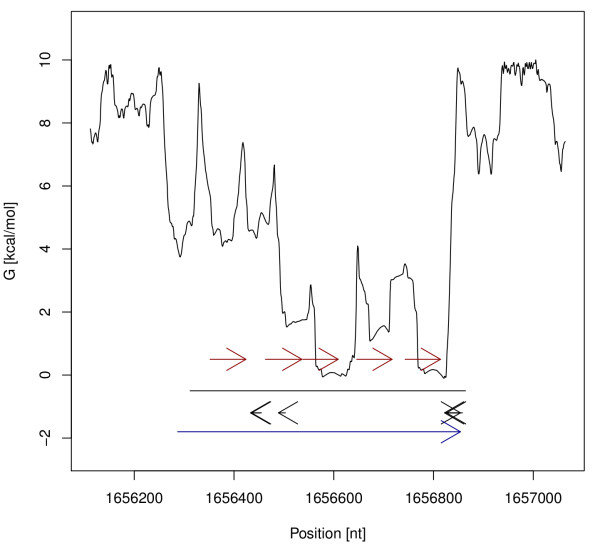
**Transcription feature plot of a tRNA locus**. Transcription feature plot of a predicted ncRNA transcript (blue arrow) covering a locus containing several tRNAs (red arrows). For a detailed legend see figure 2.

Transcript prediction for tRNA loci is also challenging, because they are often transcribed polycistronically. In many cases NOCORNAc was still able to predict the transcript correctly (see Figure 8 for an example). However, only about 50% of all tRNAs have been correctly classified as transcripts by NOCORNAc. The ability of the program to detect tRNAs could be improved by considering their specific properties. On the other hand we designed NOCORNAc to predict ncRNA transcripts in general. There are other programs that specifically aim at the prediction of tRNA genes, such as tRNAscan-SE [65].

To demonstrate NOCORNAc's functionalities we have applied it to characterize non-coding RNAs in the genome of *S. coelicolor*. NOCORNAc correctly predicted over 75% of the known ncRNA transcripts, and classified over 90% of the cis-regulatory motifs correctly. The identification of intergenic ncRNAs in *S. coelicolor *has been reported in previous studies. Pánek, *et al*. found 32 ncRNAs [48], of which we detect 15. Of the 9 ncRNAs that have been found by Swiercz, *et al*. [49] we detected 2. A comparison to SIPHT, a commonly used tool for bacterial ncRNA transcript prediction in intergenic regions, revealed that on *S. coelicolor *NOCORNAc is not only competitive but slightly better with respect to ncRNA genes and the sensitivity for tRNA genes is even significantly higher. Altogether SIPHT detected more than twice as many intergenic ncRNA transcripts in comparison to NOCORNAc, which might be due to the fact that SIPHT uses known transcription factor binding sites (TFBS) for the promoter region prediction, which are not sufficiently available for *S. coelicolor*, therefore possibly resulting in a larger number of false positive predictions in this organism. NOCORNAc is superior in its general applicability, since it can always use information of promoter signals computed by the SIDD model, while TFBS data is often insufficiently available for many bacteria.

In a previous study, transcriptomic time-series data of unprecedented resolution were used to study the metabolic switch of *S. coelicolor *and precisely profile expression changes and allocate them to specific points of time during growth [51]. In that study a custom design Affymetrix microarray was used that contained probes not only interrogating protein-coding genes but also predicted asRNAs regions as well as intergenic regions. Using that data thus allows not only to validate our predictions but also to compare the expression profiles of asRNAs with the protein-coding genes. Our analysis reveals that ncRNAs show similar complex expression dynamics as the coding genes, suggesting that they are involved in the same biological processes. Interestingly, antisense RNAs often showed a high expression correlation with their respective antisense gene. However, for those predicted elements for which no significant expression was detected we are not able to decide if they are false positive predictions or if they can be expressed under different conditions. As the proteome of the samples of the time-series is also currently analysed, we will integrate this data with the transcriptomic data to infer hypotheses about the potential function of the predicted ncRNA transcripts for which an expression was detected.

## Conclusion

With NOCORNAc we provide a program for the prediction of ncRNA transcripts to complement either *in silico *predictions of functional ncRNA loci or experimentally derived loci of expressed ncRNAs. A genome-wide expression study integrating the results of the application of NOCORNAc to *Streptomyces coelicolor*, indicated highly interesting expression dynamics of ncRNAs.

Determining the function of ncRNAs is the major challenge following their computational prediction and experimental validation. Although there are first high-throughput methods giving rise to the functional potential of ncRNAs [66], the experimental assessment of functionality usually concentrates on single elements. Therefore, we integrated approaches in NOCORNAc allowing the generation of hypotheses about the putative functionalities of the predicted elements. This includes, for example, the prediction of RNA-RNA interactions with mRNAs of protein-coding genes, which can provide hints about the potential regulatory function of the ncRNAs. A first application of this method to a subset of ncRNA transcripts predicted in *S. coelicolor *suggests that ncRNAs might even act as regulators in important metabolic processes such as antibiotic production.

## Authors' contributions

AH implemented NOCORNAc and performed the data analyses. Both authors designed the study, and wrote the manuscript. All authors read and approved the final manuscript.

## Supplementary Material

Additional file 1 **GFF file containing all results of the application of nocoRNAc to the genome of S. coelicolor**. This file is intended to be loaded into a genome browser or other programs processing chromosomal annotations. Click here for file

Additional file 2 **Table of all predicted ncRNA transcripts in the genome of S. coelicolor**. This table (xls format) contains locus information on all predicted ncRNA transcripts in the genome of *S. coelicolor*. Additional information like the RNAz region in which the transcript was predicted as well as the strength of the SIDD site and terminator are provided. Click here for file

Additional file 3 **Expression value table of putative ncRNA transcripts**. The table contains expression data of the 403 putative ncRNA transcripts for which the expression was measured at 32 time points along the growth curve of *S. coelicolor *under phosphate limited conditions. Click here for file
